# Public involvement in health and social sciences research: A concept analysis

**DOI:** 10.1111/hex.12825

**Published:** 2018-08-29

**Authors:** Mel Hughes, Catherine Duffy

**Affiliations:** ^1^ Department of Social Work and Social Sciences Bournemouth University Bournemouth UK

**Keywords:** co‐production, health, participatory research, PPI, public and patient involvement, social sciences, social work, user‐controlled research, user‐led research

## Abstract

**Background:**

Research funding bodies have significantly increased emphasis on the need for public involvement in research with the requirement to evidence effective methods and approaches to achieving this. Specific definitions and approaches within published research remain tokenistic and vague.

**Objective:**

The concept analysis explores and clarifies the nature and meaning of public involvement in health and social sciences research and identifies operational definitions which can be used to guide, develop and evaluate public involvement in research activity.

**Search strategy:**

A literature search was conducted using online databases. Systematic literature reviews and broader studies on the impact of PPI were included as was grey literature such as guidance from INVOLVE and research funding bodies. Limits were set to papers published in the last 10 years and in the English language. A concept analysis framework adapted from Rodgers (Concept Development in Nursing: Foundations, Techniques and Applications. London, UK: Saunders; 2000) and Walker and Avant (Strategies for Theory construction in Nursing. Boston, MA: Pearson Prentice Hall; 2005) was applied.

**Main results:**

Five operational definitions were developed from the concept analysis: undefined involvement; targeted consultation; embedded consultation; co‐production; and user‐led research. Typical examples of each approach were identified from the literature. Defining attributes included having clear and agreed meaning and purpose for any involvement; reciprocal relationships; and value and recognition of the expertise of all those involved.

**Conclusions:**

The authors argue the need for researchers to more explicitly incorporate and evaluate details of approaches used. Impact of public involvement on a research study should be identified when reporting on findings to prevent tokenistic practices where involvement is viewed as secondary to the core research process.

## 1 INTRODUCTION

There has been an increasing emphasis in recent years on the need for meaningful public involvement in all stages of the research cycle from shaping the health and social science research agenda to influencing what, why and how research is conducted and disseminated.^1^, ^2^, ^3^ Funding bodies such as the Wellcome Trust, the Economic and Social Research Council (ESRC), Alzheimer's Research UK, Cancer Research UK and the National Institute for Health Research (NIHR) have significantly increased their emphasis on the need for public involvement in research. Funding bids must evidence effective methods and approaches to achieving this. There is a concern, however, that one of the main reasons for public involvement in research is political mandate^4^ based on neo‐liberal, consumerist models,^1^, ^2^ which can be satisfied with tokenistic participation.

As a University partnership for Public Involvement in Education and Research (PIER partnership), our role has been to promote and support best practice and to help embed a culture of meaningful involvement in research in ways which increase impact. Our experience has been that researchers are often aware of the need to involve the public in research but not always of how or why.

Research impact is defined by the ESRC as “the demonstrable contribution that excellent research makes to society and the economy.” Impact of PPI in this analysis refers to how involvement enhances the capacity of a research study to achieve academic, economic and societal impact, that is, by making a study more relevant; as well as the perceived impact of the process on those involved. Exploration of the literature, including research papers where public involvement is claimed, shows that there is often a lack of detail regarding what public involvement entailed and what impact it had. A search on participatory research, for example, which identified 86 results found that only ten had specific details on how people were involved and the impact of this on the research process and outcomes.

The risk of not providing explicit examples and evaluations of different approaches when publishing research is that involvement remains at a tokenistic level and concepts of meaningful involvement and measures of impact remain vague. McLaughlin^5^ argues that too often positive outcomes are suggested in research purely on the basis of service users having contributed to the research. As Roy^6^ suggests, participatory research does not automatically guarantee better data or outcomes. The purpose of this study is to explore and clarify the concept of public involvement in research or the more commonly used term public and patient involvement (PPI). We identify operational definitions of different approaches as part of the process of identifying and developing involvement which has a clear purpose, maximizes impact and is meaningful for all involved.

### 1.1 Concept analysis

A concept analysis is a process to guide the exploration of a concept that may be vague or ambiguous. According to Knafl and Deitrick,^7^ concept analysis “entails the systematic examination of the attributes or characteristics of a given concept for the purpose of clarifying the meaning of that concept.” Whilst originating in mathematics, a number of concept analysis methods are now used across research disciplines and are commonly used in nursing science and education.^8^, ^9^ Whilst a concept analysis is often used to explore new and underdeveloped concepts and theories, it can also be used to clarify and define concepts which are open to individual interpretation, multiple truths and subjectivity.^10^

## 2 METHOD

We adapted Walker and Avant's^11^ concept analysis model to develop a framework for analysing the concept of public involvement in research. We used this specifically as a framework for exploring published research literature where claims of PPI were made. Rather than constructing model, borderline, related, contrary, invented and illegitimate cases to exemplify the concept, we deviate from the Avant and Walker model by incorporating model cases identified from the literature. This draws on Rodger's^12^ contextual model of concept analysis which seeks to identify rather than construct cases. The framework used was:

- Determine the aims of the analysis
- Clarify the meaning and nature of the concept
- Identify multiple uses of the concept and develop operational definitions
- Determine the defining attributes and characteristics
- Identify model cases from the literature to exemplify these in practice
- Identify antecedents and consequences for effective involvement to take place
- Define empirical referents (ways the concept can be observed and measured).

Boolean searches of terms incorporating combinations of public, patient, involvement, PPI, user‐led, co‐production, user‐controlled; participat*; combined with: research; and health, social work and social sciences, were conducted using online databases such as ASSIA, British Nursing Index, Capacity Builder and CINAHL, along with searches of the INVOLVE evidence library. Limits were set to papers published in the last 10 years in the English language. We excluded papers which related to public engagement (sharing findings) rather than involvement. Systematic literature reviews and broader studies on the impact of PPI were included as was grey literature such as guidance from INVOLVE and research funding bodies. Snowballing (searching through reference lists) was used to identify more case examples. Papers were analysed using a simple standardized evaluation questionnaire we devised which identified: whether PPI was claimed; methods of PPI used (what and how); defining attributes and characteristics, reported outcomes of using the approach; and identified benefits and barriers.

From this analysis, defining attributes were identified to clarify the meaning and nature of the concept of public involvement in research. Five operational definitions were created to exemplify the concept. These were as follows: undefined involvement; targeted consultation; embedded consultation; co‐production; and user‐led research. Case examples were identified from the literature to demonstrate practical applications of each definition.

## 3 FINDINGS

### 3.1 Aims of the analysis

The aim of the concept analysis was to explore and clarify the nature and meaning of public involvement in health and social sciences research and to develop operational definitions which can be used to guide, develop and evaluate public involvement in research activity.

### 3.2 Clarify the meaning and nature of the concept

Integral to a concept analysis is the process of exploring how terminology is currently used within the literature and in practice and to clarify meanings that can be vague or ambiguous. To conduct the concept analysis, we adopted INVOLVE's definition of patient and public involvement. INVOLVE are a government‐funded national advisory group that supports greater public involvement in NHS, public health and social care research in England. They define public involvement as “research being carried out with or by members of the public rather than to, about or for them”.^13^ They suggest public involvement can include consultation, collaboration and user‐controlled research involving the public (including patients, potential patients, carers and people who use or represent people who use health and social care services), being co‐applicants on research projects, identifying research priorities, being members of advisory groups, commenting on research materials, undertaking interviews and undertaking research.^13^

Public involvement is our preferred term given its broad and inclusive definition although we acknowledge that public and patient involvement (PPI) is more commonly referred to within the papers being reviewed.

### 3.3 Determine the defining attributes and characteristics

A defining attribute identified from the literature was that public involvement should have a clear and agreed meaning and purpose. Brett et al^14^ conducted a systematic review (66 studies) to map the impact of PPI on health and social care research. They found that effective PPI enhanced impact at all stages of a study leading to the development of user‐focused research objectives; user‐relevant research questions; user‐friendly information, questionnaires and interview schedules; more appropriate recruitment strategies for studies; and consumer‐focused interpretation of data and enhanced implementation and dissemination of study results. Dudley et al^15^ looked at the impact of PPI in randomized clinical trials. Over half of researchers, they interviewed thought it had made a difference by influencing aspects of the trial or how they, as the principal investigator, thought about the trial. The level of impact was identified as dependent on the clarity of goals and plans for PPI and the quality of the relationships between those involved. Advisory groups and trial management groups had a greater impact than the more removed, steering and oversight groups. Evidence shows that involving the public in the development of research topics, design and dissemination, impacts on how research is conducted and ensures that research is relevant, participant friendly, ethically sound and improves outcomes for patients and service users.^16^

How people are involved in research was also a defining attribute, in particular, the relationship between those involved. McKenna^17^ explored the reciprocal relationships between impact and PPI in mental health nursing studies concluding that “the latter positively influences the former.” Shippee et al^18^ who conducted a systematic review of 41 sources identified that important factors within PPI included patient and service user initiation, reciprocal relationships, co‐learning and re‐assessment and feedback. Involvement can have direct benefits for the participants themselves and as such can enhance social capital.^19^ Fudge et al^20^found that research involvement gave participants a sense of purpose and satisfaction in the knowledge that they are affecting change, as well as involvement increasing their own knowledge, skills and self‐confidence. Staley^21^ completed a review on the outcomes of using PPI and found that public involvement has the most impact when people have been included throughout the whole process rather than at certain stages.

A further defining attribute or characteristic was the value and recognition of the expertise of those involved. In the published literature, this was often referred to in relation to valuing and giving equal weighting to the contribution of those involved but also included the provision of support, training, supervision and financial remuneration to ensure full participation. Members of the public should be paid for their time and expertise and be supported and trained to participate fully in their role.

### 3.4 Identify multiple uses of the concept and develop operational definitions

From the analysis of the literature, five operational definitions were identified. The aim was to exemplify the multiple uses of the concept of public involvement in research along with case examples to demonstrate practical applications. These were undefined involvement; targeted consultation; embedded consultation; co‐production; and user‐led research. The five operational definitions are presented here along with a number of model cases identified from the literature search.

#### 3.4.1 Undefined involvement

A research study which is planned, designed and conducted without consultation or involvement from the public or where the public involvement is claimed but not explained or evaluated. Typically, those with lived experience such as service users, patients, carers, end users or benefactors are only involved as research participants, respondents or research subjects. This model can be characterised as research done ‘to’ people rather than ‘with’ people.

Most papers identified in the literature, which claimed to use PPI or public involvement in the research process, fell within this operational definition; a finding which will be discussed later.

#### 3.4.2 Targeted consultation

Involvement where members of the public, particularly those with relevant lived experience, are contacted and consulted on aspects of the research study. They may be approached to provide feedback on a plain English (lay) summary or the wording of a research survey or questionnaire; or to comment on or provide support for a research proposal. Typically people are already active in research or service user or patient groups with which the researcher has links or are part of a community organisation or online forum through which they are approached. This model can be characterised by the extent of the involvement which is limited to specific requests and tasks and where members of the public are not otherwise involved in the nature or design of the study. Those involved, may not receive much information regarding subsequent progress, outputs or impact.

##### 3.4.2.1 Examples from the literature review of targeted involvement

Knapp et al^22^ conducted a randomized control trial to test whether involving the public in the design of a public information sheet made it easier for potential research participants to read and understand all aspects of a clinical trial. One group received a public information sheet written by the researcher, and the other group, a sheet which had been revised following public feedback; 66% of participants were able to understand all aspects of the trial after reading the revised version in comparison with 15% after reading the researcher's version; 87% of participants stated they preferred the revised version. In this example, targeted involvement ensured that more participants were able to make an informed decision regarding whether they wished to participate in the study. The researchers concluded that the original un‐revised version would not have supported genuine informed consent for the study.

Boote et al^23^ provide a case study account of when an academic‐led and health practitioner supported idea for a research study was not supported by stroke survivors and carers who were asked to contribute to its development. The proposed funding bid was abandoned as a result of stakeholder views being listened to and valued. The researchers reflect on how embedded involvement from the start may have led to the development of a viable research study.

#### 3.4.3 Embedded consultation

Involvement where members of the public with relevant lived experience, are consulted with regularly throughout the research cycle from giving feedback on research ideas and proposals through to the dissemination of findings. Typically involvement includes service user or lay representation on research steering or advisory groups; regular consultation with a specialist service user advisory group or user led organisation; or methods of consulting with a range of people at different stages of a study. This model can be characterised by the regularity and range of methods for consultation. It is strengthened when involving a number of people with a range of views, experiences and perspectives and when not relying on one person or lay representative. In this model, the research team still has ownership and control over the research study but engages in meaningful consultation with others.

##### 3.4.3.1 Examples from the literature review of embedded consultation

An evaluation report of Patient and Public Involvement in the UK Clinical Research Collaboration (UKCRC)^24^ reports on a pilot where nine patient and public members were recruited to research advisory groups. Their roles were to attend and participate in the UKCRC board or board subgroups; contribute to discussions; and assist each group in understanding some of the perspectives of patients and the public that were relevant to the work of the group. Evaluation identified that by contributing to discussions, patient members made a difference by keeping discussions grounded; promoting issues or questions which members believe would be important to patients and the public and bringing in knowledge from other related experience. It was acknowledged, however, that it was difficult to judge the precise impact that one or two people will have had on the outcomes of group discussions.

#### 3.4.4 Collaboration and co‐production

Members of the public with relevant lived experience, are involved as members of the research team as researchers/co‐authors or in ways where they contribute to key decisions regarding research processes and findings. Typically this includes people contributing to decisions such as the tools used, choice and wording of research questions, how data are analysed, how research findings are presented and how research might be implemented. It may also involve writing plain English (lay) summaries, contributing as co‐authors and being part of a steering group. This model is characterised by the reciprocal nature of the relationships and collaborative processes involved, even when participants undertake different roles based on their areas of expertise. For collaboration to work and for decision making to be shared appropriately, sufficient training, supervision and support is provided.

##### 3.4.4.1 Examples from the literature review of co‐production and collaboration

McKevitt et al^25^ reflected on a pilot study exploring the personal costs of stroke to individuals and families. Need for the study was identified by members of their Stroke Research Patient and Family Group. Whilst the study was led by professional researchers, members of the group and professional researchers worked together on the investigation. This included guided conversations, interviewing each other and development of a questionnaire. The researchers analysed the data and shared this with the group. Mckevitt et al^25^ reflect on the notion of power as they were aware that they led the research process and made suggestions with which the group agreed.

Liabo^26^ conducted a systematic review which considered how looked after children could be supported to stay in school. Twenty care leavers aged 16‐24 were involved with the literature search and analysis although the number fluctuated as the young people could decide how involved they wished to be at each stage. The young people were provided with training to enable them to fully contribute. They were involved in deciding on a question, search terms, filtering articles; language to be used within the literature search and how the review was written up. The young people had a stronger say on topic‐related decisions, whereas the final decision on technical reviewing decisions was to be made by the researcher. Liabo concluded that involving the young people increased the relevance of the research question, articles used and the subsequent analysis. It improved the internal validity of the study by making research decisions more transparent and accountable and the external validity by ensuring the research was more relevant to the field.

Loughran and McCann^27^ conducted a community participatory research inquiry which investigated drug problems in three communities. Service user and community members were involved throughout the research process. Researchers maintained responsibility for overall project management at the request of the members. Service users supported the researchers in recruiting participants, running focus groups and analysing the findings. Loughran and McCann^27^ identified that service users brought different views and experiences to the study and analysis of the findings, enabled them to engage more participants, made the study more accessible to participants and used local knowledge and contacts to gather data that the researchers would not have had the ability to find. Whilst the intention had been for the research to be user led, the need for flexibility and enabling people to contribute in ways they were comfortable was acknowledged. The researchers argued that service users can make valuable contributions without necessarily becoming researchers themselves.

In a different model of collaboration, Williamson et al^28^ reviewed the impact of recruiting two older volunteer researchers to research assistant roles in a study exploring loneliness and isolation among older people. Researchers were of a pensionable age and engaged in all aspects of the research process as full members of the research team. They received training in research methods to enable them to engage in decisions regarding the research design and were involved in peer interviews of other older people. Academics found that working in partnership with the volunteers significantly improved the quality, validity and relevance of the study. Volunteers refined the wording of recruitment flyers and removed the use of “smileys” which they viewed as patronizing. They wrote their own interpretation of findings which contributed to the final study. Participants reported feeling more comfortable due to the presence of researchers of a similar age. The academics acknowledge challenges regarding the skill set of the volunteers at the start of the process. One volunteer reported feeling negatively at the start, as the language was too academic and she did not understand until “they started talking about what I understood.”

#### 3.4.5 User‐led research

Members of the public with relevant lived experience, academics and practitioners work together systematically across all areas of the research cycle. People with lived experience are supported to take the lead in directing the nature and direction of a research study. Typically, people with lived experience are involved in generating ideas, proposals, funding bids, publishing and presenting the findings and are likely to be involved in conducting the research by interviewing participants or facilitating focus groups. This model is characterised by the shift in balance of control to the people with lived experience. Members may engage in the study in different ways depending on their areas of expertise and experiences but each role is given equal value and weighting.

##### 3.4.5.1 Examples from the literature review of user‐led research

Littlechild et al^29^ recruited 22 co‐researchers (11 service users and eleven carers including people with Dementia and from Black and Minority Ethnic communities). The participatory research project focused on older people's experiences of transitions between care services. Co‐researchers received training and payment and were involved throughout the study including designing the research method and tools, recruiting and interviewing participants, identifying key themes during analysis and the dissemination of findings.

Following the study, Littlechild et al^29^ interviewed the co‐researchers, academic researchers and representatives from statutory and voluntary organizations to identify what impact involving the co‐researchers had on the study outcomes. The user‐led model was recognized as having improved recruitment, particularly from marginalized groups. It had given an authenticity and persuasiveness to the findings as they had been identified and shared by people with that experience. Co‐researchers suggested that participants felt more comfortable opening up about their lives due to shared experiences, informal style, shared language or proximity in age. During analysis, co‐researchers felt that their experiences ensured that they were aware of the significance of certain issues and ensured that these issues were noted during the dissemination of their findings. Academics did raise some concerns regarding possible bias in interviews (seeking out experiences which matched their own) or of missing issues which the academic felt to be of significance. Whilst adequate training is essential, they suggest that this should not be to the extent where it prevents the unique perspectives and approaches that involving the public can bring.

Finally, Pitt et al^30^ developed user‐led research on recovery for people with psychosis. Two service users conducted the research interviews with research supervision conducted by a clinical psychologist with experience in data collection and analysis. A steering committee of service users identified the topic and contributed to the design and analysis ensuring a broader user perspective. Benefits of this approach were identified as enhancing the choice of methodology, research design, insight into participants’ subjective experiences and insider perspectives.

### 3.5 Identify antecedents and consequences for effective public involvement in research to take place

We identified positive outcomes from meaningfully involving members of the public with lived experience, in all stages of the research process. There were clear antecedents (events that must occur prior to the occurrence of the concept) and consequences (events that must occur as a result)^11^ in all of the models. These included the need for:

- Clear goals to be identified to clarify the purpose of the involvement
- Sufficient preparation, training, support, supervision and financial remuneration to be provided to enable the public to fully contribute and undertake the roles required.
- Reciprocal relationships to be established where all involved can benefit
- Involvement and collaboration with support organizations such as in the independent, voluntary and private sector
- Members of the public to be able to contribute in different ways
- Different approaches, perspectives, skills, styles and contributions to be valued
- Academic and practice researchers to be open to relinquishing and sharing control to facilitate new ways of working.

### 3.6 Define empirical referents

Empirical referents in a concept analysis are ways in which the concept can be observed and measured. Researchers have suggested the need for agreed tools in order to measure the impact of public involvement across different stages of the research cycle and to identify the most effective approaches.^14^, ^15^, ^31^, ^32^

Adaptation of Tew et al's^33^ ladder of involvement (adapted from Arnstein's 1969 ladder of citizen participation) was considered in this concept analysis, as a measurement tool and framework. Advocates of user‐led research (the pinnacle of a ladder model) argue that service user leadership in research is the most effective way of achieving change.^2^, ^3^ Callard and Rose^2^ and Rose, Carr and Beresford^3^ argue that challenging hierarchies of power leads to the development of new perspectives, credible and legitimate knowledge^3^ and the transformation of concepts.^2^ INVOLVE,^34^ however, advise against viewing approaches to involvement as a hierarchical framework. Ladder models have received criticism for their emphasis on power rather than on quality of relationships, parity of participation and impact.^1^, ^32^ Davies et al^1^ argue that such models can exclude the most vulnerable and limit diversity. Tritter and McCallum^32^ suggest that users having agency to shape the nature of their own involvement leads to more meaningful change. South et al^31^ conducted 19 interviews with researchers and patients involved in PPI and concluded that utilizing a range of models increases impact.

The proposed empirical referents identified in this study therefore seek to identify best practice as a way of measuring effective and meaningful public involvement, rather than being based on a hierarchical framework. Researchers are encouraged to view the operational definitions, typical cases and antecedents identified within this concept analysis when considering which approaches will achieve the most significant benefits, outcomes and impact.

## 4 DISCUSSION

Conducting a concept analysis has sought to clarify the meaning and nature of public involvement in research which in practice can be tokenistic, undefined and vague. Identifying specific models from published research papers has been challenging. Model cases of embedded consultation, for example, were difficult to find within the academic literature, despite being the most prevalent model identified through online searches. A review of grey literature identified many examples of universities, research centres and hospital trusts having public or patient advisory groups attached to specific research studies and trials. These were often in specific geographical locations or in relation to specific health conditions and often with support and funding from the NIHR. Whilst there were some published evaluations of the impact of these models, few were subjected to peer review.

Developing a sound evidence base regarding public involvement in research and identifying what produces positive experiences, outcomes and impact is challenging. This is due to the nonstandard and nonempirical nature of much of the literature^14^, ^18^, ^35^, ^36^, ^37^ and the difficulty in isolating the direct action which causes a specific outcome.^37^ We identified that this is also influenced by a lack of explicit reporting of how public involvement is undertaken when publishing research findings.

Staley^21^ is critical of the restrictive styles of some peer‐reviewed journals which do not facilitate descriptions of the involvement process. The NIHR^38^ suggest that researchers write separate papers on studies which involve PPI to allow other researchers to learn from these experiences. Much of the literature analysed in this concept analysis resulted from this type of paper as they gave detailed examples and outcomes of using specific models of PPI. Whilst welcomed, this fails to acknowledge the impact of public involvement on the research findings if not incorporated into papers reporting on the outcomes of primary research. This further enforces the acceptance of tokenistic practices where involvement is viewed as secondary to the core research process.

It is clear from this concept analysis and the approaches and cases identified, that the greater and more meaningful the level of involvement the more likely there are to be positive outcomes for all involved. This is not to say that the public need to be involved at every stage for a study to be a relevant or of good quality. NIHR^38^ advise that as each study is different, PPI should be used in areas believed to be the most beneficial to that particular study. Participation in studies can range from no public involvement at some stages to being user‐led at others. Funk et al^39^ conducted a participatory research study with street‐involved young people and wished to find out factors that prevented this demographic from injection drug use. Initially, young people were consulted on the research. Over the course of the project, they engaged in team building exercises which increased how comfortable they were with the researchers and their participation increased. Whilst this unplanned development had implications for time and money, it led to the research analysis and findings being more relevant to the level of involvement being directed by the young people involved. Levels of involvement can evolve therefore as a study develops.

## 5 CONCLUSION

Significant growth in involvement activity has led to many claims of public involvement some of which remain at a tokenistic level. There is, however, a wealth of information and guidance which supports good practice in public involvement and increasing evidence of the impact this can have on all stages of the research cycle. The emphasis of this study has been to draw on these to develop operational definitions and examples from which researchers, including ourselves, can identify best practice.

From the literature, we identified that involving people in recruitment and in developing materials such as participant information sheets can lead to increased and more diverse recruitment including those from marginalized groups. Involvement in research design can lead to the development of research tools which are more relevant and easier to understand. Involvement in data collection such as conducting interviews and focus groups affects the dynamics of the relationship between participants and researcher making it a more positive experience. Involvement in the analysis of the findings can lead to a broader interpretation of what is relevant and of significance for specific user groups than if just completed by an academic or practitioner researcher. Defining characteristics of meaningful and effective involvement include having a clear and agreed meaning and purpose for any involvement; reciprocal relationships; and value and recognition of the expertise of all those involved. Recommendations for the future are for authors to more explicitly incorporate the details and impact of public involvement on the research study when reporting on findings.

## CONFLICT OF INTEREST

There are no conflicts of interest.
